# Association of Diabetic Retinopathy and Diabetic Kidney Disease With All-Cause and Cardiovascular Mortality in a Multiethnic Asian Population

**DOI:** 10.1001/jamanetworkopen.2019.1540

**Published:** 2019-03-29

**Authors:** Charumathi Sabanayagam, Miao Li Chee, Riswana Banu, Ching-Yu Cheng, Su Chi Lim, E. Shyong Tai, Thomas Coffman, Tien Y. Wong

**Affiliations:** 1 Singapore Eye Research Institute, Singapore National Eye Centre, Singapore; 2 Ophthalmology and Visual Sciences Academic Clinical Program, Duke-NUS Medical School, Singapore; 3 Khoo Tech Puat Hospital, Singapore; 4 Department of Medicine, Yong Loo Lin School of Medicine, Singapore, National University of Singapore, Singapore; 5 Cardiovascular and Metabolic Disorders Program, Duke-NUS Medical School, Singapore

## Abstract

**Importance:**

The association of diabetic microvascular complications such as diabetic retinopathy (DR) and diabetic kidney disease (DKD) with mortality in populations is not clear.

**Objective:**

To examine the association of DR and DKD separately and jointly with all-cause and cardiovascular disease (CVD) mortality in a multiethnic Asian population.

**Design, Setting, and Participants:**

A population-based cohort study was conducted including 2964 adults between the ages of 40 and 80 years with diabetes who participated in the Singapore Epidemiology of Eye Diseases study (baseline, 2004-2011). Data analysis was performed from January to May 2018.

**Exposures:**

Diabetic retinopathy ascertained from retinal photographs and DKD from estimated glomerular filtration rate.

**Main Outcomes and Measures:**

All-cause and CVD mortality obtained by linkage with the National Registry of Births and Deaths until May 2017.

**Results:**

Of the 2964 adults (mean [SD] age, 61.8 [10.0] years; 1464 [49.4%] female; 592 Chinese, 1052 Malay, and 1320 Indian), 29.9% of the participants had DR, while 20.7% had DKD. Over a median (interquartile range) follow-up of 8.8 (7.2-11.0) years, 610 deaths occurred (20.6%), of which 267 (9.0%) were due to CVD. In separate models, the multivariable hazard ratios for all-cause and CVD mortality were 1.54 (95% CI, 1.24-1.91) and 1.74 (95% CI, 1.27-2.40), respectively, for DR and 2.04 (95% CI, 1.64-2.56) and 2.29 (95% CI, 1.64-3.19), respectively, for DKD. In models including both DR and DKD, the subgroup with DKD alone (27.1% and 12.6%) followed by DR alone (6.5% and 5.2%) contributed substantially to the excess risk of all-cause and CVD mortality. Compared with those with no DR and DKD, the hazard ratios of all-cause and CVD mortality were 1.89 (95% CI, 1.40-2.57) and 2.26 (95% CI, 1.42-3.61), respectively, for DKD alone and 1.38 (95% CI, 1.03-1.86) and 1.64 (95% CI, 1.06-2.56), respectively, for DR alone. Hazard ratios for all-cause and CVD mortality were 2.76 (95% CI, 2.05-3.72) and 3.41 (95% CI, 2.19-5.32), respectively, for those with both DKD and DR. The relative excess risk associated with the interaction was 0.49 (95% CI, −0.29 to 1.27; *P* = .20) for all-cause mortality and 0.51 (95% CI, −0.83 to 1.85; *P* = .50) for CVD mortality.

**Conclusions and Relevance:**

These results suggest that risks of all-cause and CVD mortality were significantly higher in those with DKD and DR, but DKD was more strongly associated with excess risk. The findings underscore the importance of early identification and close monitoring and management of patients with DR and DKD to reduce the risk of death.

## Key Points

**Question:**

Among persons with diabetes, are diabetic retinopathy (DR) and diabetic kidney disease (DKD) associated with higher risk of mortality?

**Findings:**

In this cohort study that included 2964 Asian (Chinese, Malay, and Indian) persons with diabetes followed up for a median of 8.8 years, the presence of DR was associated with higher all-cause mortality independent of presence of DKD. Persons with both DKD and DR had the highest rate of mortality, with the excess risk largely associated with DKD.

**Meaning:**

In this study, for patients with diabetes, the presence of DR and DKD were risk factors for mortality, and identification and appropriate management of patients with both conditions may reduce the risk of mortality.

## Teaser

This population-based cohort study investigates the association of diabetic retinopathy and diabetic kidney disease with all-cause and cardiovascular mortality among multiethnic Asian adults.

## Introduction

Diabetes affected an estimated 415 million people worldwide in 2015, and the number is expected to increase to 642 million by 2040, with the greatest increase expected in Asia, in particular, India and China.^1^ Diabetes is a major risk factor for cardiovascular disease (CVD) and it is well known that diabetes is associated with an increased risk of both all-cause and CVD mortality.^2,3^ With the increasing prevalence of diabetes and the aging of the population, the prevalence of diabetic microvascular complications such as diabetic retinopathy (DR) and diabetic kidney disease (DKD) is likely to increase in parallel. Diabetic kidney disease has been shown to be associated with an excess risk of premature death, in particular CVD death, in 2 recent studies conducted in the United States^4^ and Europe.^5^ Likewise, DR, a leading cause of preventable blindness, has been shown to increase the risk of all-cause and CVD death.^6,7,8^ A study^9^ in the United States that examined the concurrent association of diabetes and DKD with mortality reported that coexistence of diabetes and DKD was associated with a substantially increased mortality risk; in the absence of DKD, diabetes alone was not associated with a large increase in mortality in the US population. Tong et al^10^ reported that those with both DR and macroalbuminuria had excess risk of mortality compared with either one alone in patients with type 2 diabetes. However, it is not clear whether DR is associated with greater risk of mortality independent of DKD and whether the joint association of DR and DKD with mortality is greater than the sum of the individual associations of the two. Given the increasing prevalence of diabetes in Asia, it is imperative to understand the associations of DKD and DR with mortality and the interaction between DKD and DR and its association with mortality, in particular, mortality due to CVD.

## Methods

### Study Cohort

Data for this study were derived from the Singapore Epidemiology of Eye Diseases (SEED) study, a population-based prospective study of eye diseases in 10 033 Chinese, Malay, and Indian adults aged 40 to 80 years in Singapore. Baseline data collection started with Malay individuals (2004-2007; n = 3280),^11^ followed by Indian individuals (2007-2009; n = 3400) and Chinese individuals (2009-2011; n = 3353).^12^ Response rates for the 3 studies were 78.7%, 75.6%, and 72.8%, respectively. The detailed methods of the SEED study have been published elsewhere.^13^ As all 3 studies followed the same methods and were conducted in the same study clinic, we combined the 3 populations and included only those with diabetes (n = 2964) for the present study. Diabetes was defined as random glucose level greater than or equal to 200 mg/dL (to convert to millimoles per liter, multiply by 0.0555), glycated hemoglobin (HbA_1c_) greater than or equal to 6.5% of total hemoglobin, self-reported physician-diagnosed diabetes, or use of antidiabetic medication. We defined type 1 diabetes as development of diabetes before the age of 30 years. For DR analysis, after excluding those who had missing data for fundus photography and other covariates, the final sample size was 2880; for DKD, after excluding those with missing estimated glomerular filtration rate (eGFR) and other covariates, 2858 were included in the final analysis. We assessed diabetic nephropathy (DN) separately based on albuminuria; however, data on albuminuria were not available in two-thirds of Malay participants. Hence, we did not include albuminuria in the current analysis. This study was performed in accordance with the tenets of the Declaration of Helsinki^14^ and ethics approval was obtained from the Singapore Eye Research Institute institutional review board. Written informed consent was provided by participants. This study followed the Strengthening the Reporting of Observational Studies in Epidemiology (STROBE) reporting guideline for cohort studies.

### Assessment of Outcomes

Information on all-cause and CVD-related mortality was obtained by linkage of SEED baseline data to the National Registry of Births and Deaths. Outcomes were assessed up to the date of death or May 31, 2017, whichever occurred first. As it is mandatory to report all deaths occurring in Singapore, the registry was 100% accurate in capturing all reported deaths. Deaths related to CVD included death due to cardiovascular causes, including acute myocardial infarction, coronary heart disease, hypertensive and other heart disease, heart failure, and stroke (*International Classification of Diseases, Ninth Revision*, codes 390-459 and *International Classification of Diseases, Tenth Revision*, codes I00-I99).

### Assessment of DR

After pupil dilation, 2-field color photographs (Early Treatment for Diabetic Retinopathy Study [ETDRS] field 1, centered on the optic disc, and field 2, centered on the fovea) for both eyes of all participants were obtained using a digital retinal camera (Canon CR-1 Mark-II Non-mydriatic Digital Retinal Camera). We considered DR to be present if any characteristic lesion was present, including microaneurysms, hemorrhages, cotton wool spots, intraretinal microvascular abnormalities, hard exudates, venous beading, and new vessels. Severity of DR was graded by trained graders at the University of Sydney, Australia, according to the modified Airlie House classification system^15^ and categorized into minimal nonproliferative DR (NPDR) (level 20), mild NPDR (level 35), moderate NPDR (levels 43-47), severe NPDR (level 53), and proliferative DR (PDR) (levels >60).^13^ Based on the severity score of the worse eye, a level greater than or equal to 20 was defined as any DR.

### Assessment of DKD

We defined DKD as an eGFR of less than 60 mL/min/1.73 m^2^ as recommended by the National Kidney Foundation’s Kidney Disease Outcomes Quality Initiative guidelines.^16^ We estimated GFR based on plasma creatinine using the Chronic Kidney Disease Epidemiology Collaboration equation.^17^ Serum creatinine was measured by the Jaffe method on the Beckman DXC800 analyzer calibrated to the isotope dilution mass spectrometry method using the National Institute of Standards and Technology reference material. Based on eGFR, DKD severity was classified into 4 groups: eGFR greater than or equal to 60 mL/min/1.73 m^2^ (reference representing normal high or mild decrease in kidney function), mild to moderate (eGFR 45-59 mL/min/1.73 m^2^), moderate to severe (eGFR 30-44 mL/min/1.73 m^2^), and severe or renal failure (eGFR <30 mL/min/1.73 m^2^).^18^

### Assessment of Other Factors Associated With Mortality

An interviewer-administered questionnaire was used to collect participants’ demographic data (age, sex, ethnicity, and education level); lifestyle characteristics (cigarette smoking and alcohol consumption); and personal history of diabetes, hypertension, and CVD (defined as self-reported myocardial infarction, angina, or stroke). Physical examination included height, weight, and blood pressure (BP) measurements. We calculated body mass index (BMI) as weight in kilograms divided by height in meters squared. Hypertension was defined as systolic BP greater than or equal to 140 mm Hg, diastolic BP greater than or equal to 90 mm Hg, or self-reported physician-diagnosed hypertension or use of BP-lowering medication. Laboratory measurements included random plasma glucose level, HbA_1c_ percentage, serum creatinine level, and total and high-density lipoprotein (HDL) cholesterol values.

### Statistical Analysis

We compared the baseline characteristics of the participants stratified by ethnicity. To examine the association of the interaction between DR and DKD with mortality (DN was not included in the main joint association model owing to a large number of missing data), we created 4 categories by combining DR and DKD: no DR and no DKD, representing background risk (reference, − −), DR only (+ −), DKD only (− +), and both DR and DKD (+ +). We plotted the incidence of all-cause and CVD mortality with 1 minus Kaplan-Meier failure function and compared survival distributions across categories of DR and DKD using log-rank tests. We calculated the absolute mortality rates in each of the 4 categories and the hazard ratio (HR) and 95% confidence intervals associated with each category compared with the reference group. We examined for interaction on additive scale using relative excessive risk due to interaction (RERI) and on the multiplicative scale using cross-product interaction terms.^19^ Additive interaction is present when the combined associations of DR and DKD alone are more than the sum of the individual associations. We calculated RERI using the formula (HR_+ +_) – (HR_+ −_) – (HR_− +_) + 1. Estimates of RERI were considered significant when 95% confidence intervals of RERI did not contain zero. We examined the risks of all-cause and CVD mortality by presence and severity of DR and DKD separately and jointly using Cox proportional hazards regression models adjusted for age, sex, and ethnicity and a multivariable model that additionally adjusted for primary school education or less, current smoking, alcohol consumption, history of CVD, BMI, hypertension, HbA_1c_ level, duration of diabetes, and total and HDL cholesterol measurements. In a supplementary analysis, we examined the risks of all-cause and CVD mortality in those without diabetes (n = 6621) and presence or absence of DR and/or DKD in those with diabetes (n = 2775). For this analysis, we compared mortality risks across 4 categories: normal glucose level (reference, defined as HbA_1c_ <5.7% and random plasma glucose <140 mg/dL in those with no history of diabetes), prediabetes (HbA_1c_ of 5.7%-6.4% and/or random plasma glucose of 140-200 mg/dL in those with no history of diabetes), diabetes without DR or DKD, and diabetes with DR and/or DKD. All analyses were performed using Stata statistical software version 13.0 (StataCorp LLC).

## Results

Of the 2964 adults with diabetes aged 40 to 80 years (mean [SD] age, 61.8 [10.0] years), 49.4% (1464) were women, 1.8% (53) had type 1 diabetes, and 98.2% (2911) had type 2 diabetes. Indian individuals were significantly greater in number (44.5% [1320 participants]) compared with Malay individuals (35.5% [1052 participants]) (difference for Indian vs Malay, 9.0%; 95% CI, 6.5%-11.5%; *P* < .001) and Chinese individuals (20.0% [592 participants]) (difference for Indian vs Chinese, 24.5%; 95% CI, 21.7%-26.3%; *P* < .001). Table 1 shows the characteristics of the participants stratified by ethnicity. In general, Malay participants were more likely to be female and had higher prevalence of primary education or less, smoking, hypertension, and DKD, including severe DKD (eGFR <30 mL/min/1.73 m^2^); lower prevalence of drinking; higher BMI, systolic and diastolic BP, HbA_1c_ levels, and total cholesterol levels; and lower eGFR. Indian participants had higher prevalence of drinking, history of CVD (including prior heart attacks), and antidiabetic medication use; higher BMI; longer duration of diabetes; and lower levels of HDL cholesterol. In addition, Indian participants had higher prevalence of any DR but lower prevalence of moderate and severe DR compared with the other 2 ethnic groups; they also had had higher prevalence of normal kidney function (eGFR >60 mL/min/1.73 m^2^) and lower prevalence of severe DKD (eGFR <30 mL/min/1.73 m^2^ occurred in 1.5% of Indian vs 3.5% of Chinese vs 5.1% of Malay individuals). Chinese participants were older, had lower prevalence of history of CVD (including prior heart attacks), and had lower BMI measurements. Over a median (interquartile range) follow-up of 8.8 (7.2-11.0) years, 610 participants (20.6%) died; 267 (9.0%) of these deaths were due to CVD (data not shown).

**Table 1.** Baseline Characteristics of 2964 Singapore Epidemiology of Eye Diseases Participants With Diabetes

Characteristic	Malay (n = 1052)	Indian (n = 1320)	Chinese (n = 592)	*P* Value
Age, mean (SD), y	62.6 (9.9)	60.4 (10.0)	63.5 (9.6)	<.001
Female, No. (%)	568 (54.0)	622 (47.1)	274 (46.3)	.001
Primary education or less, No. (%)	859 (82.0)	813 (61.8)	389 (65.7)	<.001
Current smoker, No. (%)	156 (14.9)	159 (12.1)	83 (14.0)	.12
Ever consume alcohol, No. (%)	5 (0.5)	160 (12.1)	51 (8.6)	<.001
Duration of diabetes, mean (SD) y	5.9 (8.2)	8.5 (9.4)	7.7 (9.0)	<.001
Antidiabetic medication use, No. (%)	562 (53.5)	863 (68.0)	367 (62.7)	<.001
Hypertension, No. (%)	880 (84.0)	939 (71.4)	463 (78.2)	<.001
History of cardiovascular disease, No. (%)	187 (17.9)	285 (21.7)	86 (14.5)	.001
History of heart attacks, No. (%)	112 (10.7)	184 (14.0)	56 (9.5)	.006
Body mass index, mean (SD)^a^	27.6 (4.9)	27 (5.0)	25.2 (3.8)	<.001
Systolic blood pressure, mean (SD), mm Hg	154.2 (23.4)	140.1 (19.8)	142.6 (19.5)	<.001
Diastolic blood pressure, mean (SD), mm Hg	80.1 (11.4)	77.3 (10.2)	76.7 (9.4)	<.001
Random blood glucose, mean (SD), mg/dL	178.2 (90.0)	174.6 (81.0)	176.4 (88.2)	.64
Glycated hemoglobin, mean (SD), %	8.0 (1.9)	7.6 (1.6)	7.4 (1.4)	<.001
Serum total cholesterol, mean (SD), mg/dL	216.6 (50.3)	189.5 (46.4)	193.3 (46.4)	<.001
Serum high-density lipoprotein cholesterol, mean (SD), mg/dL	50.3 (11.6)	38.7 (11.6)	46.4 (11.6)	<.001
eGFR, mean (SD), mL/min/1.73 m^2^	69.4 (23.4)	84.0 (20.2)	82.9 (23.7)	<.001
Chronic kidney disease, No. (%)	347 (34.2)	170 (13.3)	76 (13.5)	<.001
Diabetic kidney disease severity, No. (%)				
eGFR >60 mL/min/1.73 m^2^	669 (65.9)	1107 (86.7)	489 (86.5)	<.001
eGFR 45-60 mL/min/1.73 m^2^	184 (18.1)	109 (8.5)	37 (6.6)	
eGFR 30-44 mL/min/1.73 m^2^	111 (10.9)	42 (3.3)	19 (3.4)	
eGFR <30 mL/min/1.73 m^2^	52 (5.1)	19 (1.5)	20 (3.5)	
Diabetic retinopathy severity, No. (%)				
None	716 (71.0)	870 (67.6)	432 (74.4)	.003
Minimal or mild	166 (16.5)	281 (21.8)	84 (14.5)	
Moderate	70 (6.9)	71 (5.5)	34 (5.9)	
Severe nonproliferative diabetic retinopathy or proliferative diabetic retinopathy	56 (5.6)	66 (5.1)	31 (5.3)	

Abbreviation: eGFR, estimated glomerular filtration rate.

SI conversion factors: To convert random blood glucose to mmol/L, multiply by 0.0555; total and high-density lipoprotein cholesterol to mmol/L, multiply by 0.0259.

^a^ Calculated as weight in kilograms divided by height in meters squared.

eTable 1 in the Supplement presents characteristics of participants across the 4 disease groups (no DR and no DKD, DR only, DKD only, and both DR and DKD). Of the 2775 participants with information on both DR and DKD, 21.0% had DR alone, 11.8% had DKD alone, and 8.3% had both DR and DKD. Of the 4 groups, the group with both DR and DKD had higher prevalence of primary education or less, antidiabetic medication use, hypertension, and history of CVD (including heart attacks); higher levels of systolic BP; and longer duration of diabetes but lower levels of diastolic BP and lower eGFR. Those with DKD alone were older and had lower levels of random blood glucose. Those with DR alone had lower BMI, higher levels of random blood glucose, and higher HbA_1c_ percentage compared with the other 3 groups. Compared with the DKD-only group (11.6%), those with DR and DKD had significantly higher prevalence of severe DKD (20.3%) (*P* < .001). Similarly, compared with the DR-only group (11.8%), those with DR and DKD had higher prevalence of severe NPDR and PDR (30.3%).

In participants with diabetes, 29.9% (862) had DR, while 20.7% (579) had DKD. Incidence of all-cause and CVD death was significantly higher in those with DR or DKD compared with those without (Table 2). Risks increased significantly with increasing severity of DR and DKD. In separate multivariable models, DR and DKD were independently associated with both all-cause and CVD mortality. Hazard ratios for all-cause and CVD mortality were 1.54 (95% CI, 1.24-1.91) and 1.74 (95% CI, 1.27-2.40), respectively, for DR and 2.04 (95% CI, 1.64-2.56) and 2.29 (95% CI, 1.64-3.19), respectively, for DKD. In severity analyses, moderate DR (HR, 1.78; 95% CI, 1.26-2.52) and severe NPDR and PDR (HR, 2.75; 95% CI, 1.93-3.92) were significantly associated with all-cause mortality; corresponding HRs for CVD mortality were 1.95 (95% CI, 1.20-3.19) for moderate DR and 3.41 (95% CI, 2.11-5.5) for severe NPDR and PDR. With DKD, all severity categories were significantly associated with both outcomes. Hazard ratios of all-cause and CVD mortality were 1.51 (95% CI, 1.15-1.99) and 1.57 (95% CI, 1.04-2.38) for mild to moderate DKD and 6.47 (95% CI, 4.65-9.01) and 7.84 (95% CI, 4.90-12.57) for severe DKD. There was no significant interaction by ethnicity between the risks of DKD or DR and mortality (*P* for interaction >0.5 for both DKD and DR).

**Table 2.** All-Cause and CVD Mortality by Presence and Severity of DR and DKD in Those With Diabetes

Characteristics	All-Cause Mortality				CVD Mortality			
	No. at Risk (No. of Cases)	Incidence, %	Age-, Sex-, and Ethnicity-Adjusted HR (95% CI)	Multivariable HR (95% CI)^a^	No. at Risk (No. of Cases)	Incidence, %	Age-, Sex-, and Ethnicity-Adjusted HR (95% CI)	Multivariable HR (95% CI)^a^
DR^b^								
No	2018 (338)	16.8	1 [Reference]	1 [Reference]	2018 (128)	6.3	1 [Reference]	1 [Reference]
Yes	862 (242)	28.1	1.94 (1.64-2.29)	1.54 (1.24-1.91)	862 (126)	14.6	2.65 (2.07-3.40)	1.74 (1.27-2.40)
DKD^c^								
No	2265 (314)	13.9	1 [Reference]	1 [Reference]	2265 (128)	5.7	1 [Reference]	1 [Reference]
Yes	593 (265)	44.7	2.09 (1.75-2.50)	2.04 (1.64-2.56)	593 (126)	21.3	2.67 (2.04-3.49)	2.29 (1.64-3.19)
DR severity								
None	2018 (338)	16.8	1 [Reference]	1 [Reference]	2018 (128)	6.3	1 [Reference]	1 [Reference]
Minimal or mild	532 (118)	22.2	1.40 (1.14-1.73)	1.25 (0.96-1.62)	532 (53)	10.0	1.65 (1.20-2.28)	1.33 (0.90-1.96)
Moderate	175 (55)	31.4	2.38 (1.78-3.17)	1.78 (1.26-2.52)	175 (32)	18.3	3.66 (2.47-5.40)	1.95 (1.20-3.19)
Severe NPDR or PDR	155 (69)	44.5	4.12 (3.16-5.35)	2.75 (1.93-3.92)	155 (41)	26.5	6.46 (4.52-9.25)	3.41 (2.11-5.51)
DKD severity								
eGFR >60 mL/min/1.73 m^2^	2265 (314)	13.9	1 [Reference]	1 [Reference]	2265 (128)	5.7	1 [Reference]	1 [Reference]
eGFR 45-60 mL/min/1.73 m^2^	330 (114)	34.6	1.55 (1.24-1.93)	1.51 (1.15-1.99)	330 (49)	14.9	1.77 (1.26-2.50)	1.57 (1.04-2.38)
eGFR 30-44 mL/min/1.73 m^2^	172 (78)	45.4	1.96 (1.51-2.54)	1.82 (1.31-2.53)	172 (38)	22.1	2.63 (1.79-3.86)	2.11 (1.31-3.40)
eGFR <30 mL/min/1.73 m^2^	91 (73)	80.2	6.08 (4.65-7.96)	6.47 (4.65-9.01)	91 (39)	42.9	9.03 (6.15-13.27)	7.84 (4.90-12.57)

Abbreviations: CVD, cardiovascular disease; DR, diabetic retinopathy; DKD, diabetic kidney disease; eGFR, estimated glomerular filtration rate; HR, hazard ratio; NPDR; nonproliferative diabetic retinopathy; PDR, proliferative diabetic retinopathy.

^a^ Adjusted for age, sex, ethnicity, primary education or less, history of CVD, current smoking, alcohol consumption, body mass index, hypertension, total and high-density lipoprotein cholesterol, glycated hemoglobin, and diabetes duration.

^b^ *P* value for interaction for DR × ethnicity = .40.

^c^ *P* value for interaction for DKD × ethnicity = .30.

**Figure 1.**
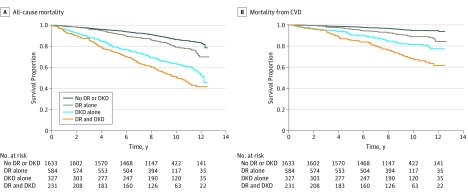
Joint Association of Diabetic Retinopathy (DR) and Diabetic Kidney Disease (DKD) With All-Cause and Cardiovascular Disease (CVD) Mortality

**Figure 2.**
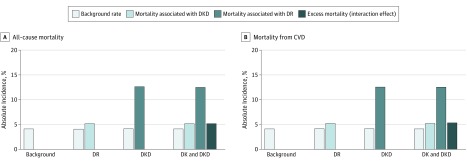
Additive Interaction of Diabetic Retinopathy (DR) and Diabetic Kidney Disease (DKD) on Absolute All-Cause and Cardiovascular Disease (CVD) Mortality Rates

Figure 1 shows the joint association of DR and DKD with all-cause and CVD mortality. Compared with controls, those with either DKD or DR had lower survival and those with both DR and DKD had the lowest survival for both all-cause and CVD mortality. Figure 2 shows the additive interaction between DR and DKD and its association with absolute rates of all-cause and CVD mortality. Of the 2775 participants with diabetes and data on DR and DKD, 58.9% had neither DR nor DKD, 21% had DR alone, 11.8% had DKD alone, and 8.3% had both DR and DKD. The absolute all-cause mortality was lowest in those without DR or DKD (background rate of 12%). The mortality rate in participants with DR was slightly increased (18.5%, ie, background rate plus additional 6.5% associated with DR), but was substantially increased in those with DKD (39.1%, ie, background rate plus additional 27.1% associated with DKD). On top of the combined mortality rate associated with diabetes, DR, and DKD, an additional 5.1% (50.7% − 12.0% − 6.5% − 27.1%) was associated with the interaction between DR and DKD. For absolute CVD mortality, the background rate was 4.2%, an additional 5.2% was associated with DR, an additional 12.6% was associated with DKD, and an additional 5.3% was associated with the interaction between DR and DKD.

In multivariable models examining the joint association of DR and DKD with mortality (Table 3), compared with those without either condition, the HRs of all-cause and CVD mortality were 1.89 (95% CI, 1.40-2.57) and 2.26 (95% CI, 1.42-3.61), respectively, for DKD alone; 1.38 (95% CI, 1.03-1.86) and 1.64 (95% CI, 1.06-2.56), respectively, for DR alone; and 2.76 (95% CI, 2.05-3.72) and 3.41 (95% CI, 2.19-5.32), respectively, for those with both DKD and DR. Other risk factors that were significant for both outcomes in the multivariable model were age, male sex, history of CVD, and HbA_1c_ percentage. In addition, Indian ethnicity was inversely associated with all-cause mortality (eTable 2 in the Supplement). No substantial additive interaction was observed for either mortality outcome with a RERI estimate of 0.49 (95% CI, −0.29 to 1.27; *P* = .20) for all-cause mortality and 0.51 (95% CI, −0.83 to 1.85; *P* = .50) for CVD mortality. Interaction between DR and DKD on a multiplicative scale was also not significant for either outcome. It is possible that the lack of additive and multiplicative association of both variables with mortality might be due to pooling together all cases of DKD and DR without regard for the disease severity. To account for severity, in a sensitivity analysis, we assessed interaction between severity categories of DR and DKD in association with all-cause mortality. Similar to the main analysis (DR and DKD) presented in Table 3, there was no significant interaction between severity of DR and DKD in association with all-cause mortality.

**Table 3.** Joint Association of DR and DKD With Adjusted All-Cause and CVD Mortality Risk

Diagnosis	All-Cause Mortality			CVD Mortality		
	No. at Risk (No. of Cases)	Incidence, %	Multivariable HR (95% CI)^a^	No. at Risk (No. of Cases)	Incidence, %	Multivariable HR (95% CI)^a^
No DR and no DKD	1633 (196)	12.0	1 [Reference]	1633 (68)	4.2	1 [Reference]
DR alone	584 (108)	18.5	1.38 (1.03-1.86)	584 (55)	9.4	1.64 (1.06-2.56)
DKD alone	327 (128)	39.1	1.89 (1.40-2.57)	327 (55)	16.8	2.26 (1.42-3.61)
Both DR and DKD^b^	231 (117)	50.7	2.76 (2.05-3.72)	231 (63)	27.3	3.41 (2.19-5.32)

Abbreviations: CVD, cardiovascular disease; DR, diabetic retinopathy; DKD, diabetic kidney disease; HR, hazard ratio.

^a^ Adjusted for age, sex, ethnicity, primary education or less, history of CVD, current smoking, alcohol consumption, body mass index, hypertension, total and high-density lipoprotein cholesterol, glycated hemoglobin, and diabetes duration.

^b^ *P* value for interaction (DR × DKD) for all-cause mortality = .80; *P* value for interaction (DR × DKD) for CVD mortality = .78

In a supplementary analysis, we compared the association of presence of diabetes and DR and DKD with absence of diabetes on mortality. In survival analysis, 5-year survival ranged from 97% in the reference group to 96% in the group with prediabetes and 95% in the group with diabetes without DR and DKD (log-rank *P* > .10) but was significantly lower in those with DR and/or DKD (84%; log-rank *P* < .001). In regression models, compared with controls, the multivariable HRs of all-cause and CVD mortality in those with DR and/or DKD were 2.12 (95% CI, 1.76-2.56) and 2.65 (95% CI, 1.97-3.58), respectively. Hazard ratios of all-cause and CVD deaths in prediabetes (HR, 0.95; 95% CI, 0.80-1.14 and HR, 0.93; 95% CI, 0.69-1.25, respectively) and diabetes without DR or DKD (HR, 1.12; 95% CI, 0.91-1.38 and HR, 1.03; 95% CI, 0.73-1.47) were not significant.

## Discussion

In a population-based sample of multiethnic Asian adults, we found that (1) the presence of DR and DKD in those with diabetes was independently associated with increased risk of all-cause and CVD mortality; (2) risk increased with increasing severity, with even mild or minimal DR and moderate DKD associated with increased risks; and (3) in joint association models, although those with both DR and DKD had the highest rate of all-cause and CVD mortality (50.7% and 27.3%, respectively), DKD alone contributed to a substantial amount of excess all-cause and CVD mortality (27.1% and 12.6%, respectively) followed by DR alone (6.5% and 5.2%, respectively), but there were no significant interactions either on additive or multiplicative scale between DR and DKD on all-cause or CVD mortality. To our knowledge, this is the first study in an Asian population that assessed the association of DR and DKD with all-cause and CVD mortality.

The mortality rate reported in the current study of 20.6% over a median follow-up of 8.8 years was high. This is consistent with a previous study^20^ that reported a higher prevalence of cardiovascular risk factors and premature deaths in rapidly transitioning Southeast Asian countries compared with countries experiencing long-standing affluence such as Japan and South Korea. It is also consistent with the 2014 World Health Organization global status report that showed premature deaths from noncommunicable diseases to be 19.6% in Malaysia, 23.1% in Indonesia, and 27.9% in the Philippines compared with 12.0% in the United Kingdom.^21^

In the current study, we found both presence and severity of DKD to be significantly associated with both all-cause and CVD mortality. Compared with participants with eGFR greater than 60 mL/min/1.73 m^2^, even those with eGFR from 45 to 60 mL/min/1.73 m^2^ had 1.51 times increased risk of all-cause mortality and 1.57 times increased risk of CVD mortality. With moderate or severe DKD, the risk increased to 6.47 times for all-cause mortality and to 7.84 times for CVD mortality. Few studies conducted in the United States and Europe have shown increased risk of all-cause and CVD mortality in those with diabetes and DKD.^4,5,9^ In a US study including insurance enrollees, Nichols et al^4^ showed that the adjusted incidence of all-cause mortality increased with increasing severity of DKD (decreasing levels of eGFR), similar to our study finding. In the MADIABETES Study^5^ in Europe, DKD was associated with 2.11 times increased risk of all-cause mortality and 1.82 times increased risk of CVD mortality.

We found severe NPDR and PDR to be associated with 2.75 times higher risk of all-cause mortality and 3.41 times higher risk of CVD mortality. A meta-analysis^8^ of published prospective studies showed the risk of all-cause mortality associated with NPDR was 1.38 (95 % CI, 1.11-1.70) and the risk associated with PDR was 2.32 (95% CI, 1.75-3.06). In the same study, participants with DR had 1.74 times increased risk of all-cause mortality for stroke. In the Reykjavik Study,^6^ those with both DR and DN had 2 times increased risk of all-cause mortality (95% CI, 1.22-3.31).

In the current study, in joint association models, beyond the background risk, substantial excess mortality was associated with DKD (27.1% for all-cause mortality and 12.6% for CVD mortality) and to some extent with DR (6.5% for all cause and 5.2% for CVD), but there was no significant excess risk associated with the interaction of DR and DKD. Furthermore, our results suggest that risk of death (including CVD deaths) in those with diabetes but without DR and/or DKD were similar to those without diabetes but increased substantially when there was DKD and/or DR. These findings are consistent with several previous studies including both type 1^22,23^ and type 2^9,24^ diabetes. In the Pittsburgh Epidemiology of Diabetes Complications Study,^22^ the 20-year risk of mortality in those with type 1 diabetes but without renal impairment during follow-up was similar to that of the general population. Similarly, in the FinnDiane Study^23^ mortality rates in individuals with normoalbuminuric type 1 diabetes were not significantly different from those of the general population. A Swedish registry-based study^24^ reported that mortality in persons with type 2 diabetes varied greatly from substantial to lower risks of death depending on age, glycemic control, and renal complications. Subgroups that were younger, had poor glycemic control, and had renal complications had excess risk. Afkarian et al^9^ found that, on the absolute scale, compared with those without diabetes and DKD (background risk = 7.7%), those with diabetes alone had 3.9% increased risk, those with DKD alone had 9.5% increased risk, and those with both diabetes and DKD had a 23% increased risk of mortality. Authors concluded that DKD in diabetes was associated with substantially increased mortality risk and, in the absence of DKD, diabetes was not associated with a large increase in mortality risk.^9^ Our findings of excess mortality in those with diabetes and DKD support results from the Swedish study^24^ and Afkarian et al^9^ and extend the previous works by evaluating the influence of DR in addition to DKD.

Strengths of our study include a multiethnic Asian population with long follow-up and information on both all-cause and CVD mortality and information on microvascular complications, including DR and DKD, which were assessed using objective measures. Our study sample is fairly representative of Singapore’s general population in terms of age distribution, housing type, and socioeconomic status according to the 2000 Singapore census.^11,12^

### Limitations

Our study has some limitations. First, our definition of diabetes was based on random glucose level, and prediabetes was based on HbA_1c_ level alone. This would have resulted in some misclassification, but the bias would be nondifferential and would be similar across both outcomes. Second, information on albuminuria, another important indicator of DKD, was missing in most of the Malay participants. Therefore, we were unable to study the association of albuminuria with risk of mortality. Third, differences in risk factor profile and follow-up duration among the 3 ethnic groups may have also influenced the association of DR and DKD with mortality. Fourth, although our sample size of 2880 participants with 580 deaths had greater than 99% power to detect HRs of 1.54 and 2.04 for association of DR and DKD, respectively, with all-cause mortality, the power to detect interaction between DR and DKD was limited.

## Conclusions

Our study findings showed that the risk of all-cause and CVD mortality was substantially higher in participants with DKD, and to some extent in those with DR. Our findings suggest that regular screening of diabetic participants for DR and DKD and close monitoring and management of these conditions may reduce the risk of all-cause and CVD death in this Asian population.

## Notes

Supplement. **eTable 1.** Baseline Characteristics of SEED Participants Across the 4 Disease Groups **eTable 2.** Association of Risk Factors With All-Cause and CVD Mortality (Joint Effect of DR and DKD Model)
