# Supplementary material for: Association of Diabetic Retinopathy and Diabetic Kidney Disease With All-Cause and Cardiovascular Mortality in a Multiethnic Asian Population
Source: JAMA Netw Open. 2019 Mar 29;2(3):e191540. doi: 10.1001/jamanetworkopen.2019.1540 (PMC6450319; doi:10.1001/jamanetworkopen.2019.1540)
Supplement: Supplement. — eTable 1. Baseline Characteristics of SEED Participants Across the 4 Disease Groups eTable 2. Association of Risk Factors With All-Cause and CVD Mortality (Joint Effect of DR and DKD Model) [file jamanetwopen-2-e191540-s001.pdf]

# Supplementary Online Content

Sabanayagam C, Chee ML, Banu R, et al. Association of diabetic retinopathy and diabetic kidney disease with all-cause and cardiovascular mortality in a multiethnic Asian population. *JAMA Netw Open*. 2019;2(3):e191540. doi:10.1001/jamanetworkopen.2019.1540

**eTable 1.** Baseline Characteristics of SEED Participants Across the 4 Disease Groups

**eTable 2.** Association of Risk Factors With All-Cause and CVD Mortality (Joint Effect of DR and DKD Model)

This supplementary material has been provided by the authors to give readers additional information about their work.

© 2019 Sabanayagam C et al. *JAMA Network Open.*

| eTable 1. Baseline characteristics of SEED participants across the 4 disease groups | | | | | |
|---|---|---|---|---|---|
| **Characteristics** | **No DR and no DKD (n=1633)** | **DR alone (n=584)** | **DKD alone (n=327)** | **Both DR and DKD (n=231)** | **P** |
| Age (years), mean SD | 59.9 (9.9) | 59.9 (9.0) | 69.0 (8.0) | 66.3 (7.8) | <0.001 |
| Gender, female | 802 (49.1) | 278 (47.6) | 150 (45.9) | 126 (54.5) | 0.206 |
| Primary/below education, % | 1038 (63.8) | 414 (71.1) | 270 (82.8) | 195 (84.8) | <0.001 |
| Current smoker, % | 256 (15.7) | 80 (13.7) | 31 (9.6) | 17 (7.4) | <0.001 |
| Ever drinking , % | 129 (7.9) | 64 (11.0) | 12 (3.7) | 3 (1.3) | <0.001 |
| Duration of diabetes (years) | 4.8 (7.1) | 11.2 (9.8) | 7.3 (8.9) | 14.4 (10.3) | <0.001 |
| Anti-diabetic medication use, % | 831 (51.8) | 450 (79.2) | 194 (60.4) | 184 (81.4) | <0.001 |
| Hypertension, % | 1157 (71.1) | 450 (77.2) | 295 (90.5) | 215 (93.1) | <0.001 |
| History of cardiovascular disease, % | 242 (14.9) | 105 (18.1) | 95 (29.2) | 78 (33.9) | <0.001 |
| Body mass index ($kg/m^2$) | 27.1 (4.8) | 26.3 (4.5) | 27.2 (4.8) | 26.9 (5.2) | 0.003 |
| Systolic blood pressure (mmHg) | 142.3 (20.1) | 146.7 (22.2) | 149.3 (23.4) | 156.2 (25.5) | <0.001 |
| Diastolic blood pressure (mmHg) | 78.6 (10.2) | 78.1 (10.5) | 77.2 (10.4) | 76.9 (12.0) | 0.027 |
| Random blood glucose (mg/dl ) | 167.4 (75.6) | 203.4 (102.6) | 163.8 (77.4) | 196.2 (102.6) | <0.001 |
| HbA1c, % | 7.5 (1.6) | 8.3 (1.9) | 7.4 (1.5) | 8.1 (1.8) | <0.001 |
| Serum total cholesterol (mg/dl) | 201.1 (46.4) | 193.3 (50.3) | 201.1 (50.3) | 197.2 (58.0) | 0.049 |
| Serum HDL cholesterol (mg/dl) | 42.5 (11.6) | 42.5 (11.6) | 46.4 (11.6) | 46.4 (15.5) | 0.20 |
| eGFR (mL/min/1.73 $m^2$) include category as well,(<60, 30-60, 30) | 87.6 (14.7) | 88.1 (17.1) | 46.2 (12.0) | 41.5 (14.0) | <0.001 |
| **eGFR stage** | | | | | |
| eGFR >60ml/min/1.73$m^2$ | 1633 (100.0) | 584 (100.0) | 0 (0.0) | 0 (0) | <0.001 |

© 2019 Sabanayagam C et al. *JAMA Network Open.*

| | | | | | |
|---|---|---|---|---|---|
| eGFR 45-60 | 0 (0.0) | 0 (0.0) | 205 (62.7) | 114 (49.4) | |
| eGFR 30-45 | 0 (0.0) | 0 (0.0) | 84 (25.7) | 70 (30.3) | |
| eGFR< 30 | 0 (0.0) | 0 (0.0) | 38 (11.6) | 47 (20.3) | |
| Heart attack (self-reported history) | 154 (9.4) | 55 (9.5) | 60 (18.5) | 59 (25.7) | <0.001 |
| **DR severity** | | | | | |
| None | 1633 (100.0) | 0 (0.0) | 327 (100.0) | 0 (0) | <0.001 |
| Minimal/ Mild | 0 (0.0) | 399 (68.3) | 0 (0.0) | 110 (47.6) | |
| Moderate | 0 (0.0) | 116 (19.9) | 0 (0.0) | 51 (22.1) | |
| Severe/ PDR | 0 (0.0) | 69 (11.8) | 0 (0.0) | 70 (30.3) | |

Data presented are frequency (percentage) or mean (standard deviation) as appropriate
P<0.0001 for all variables except random blood glucose (p=0.6)

© 2019 Sabanayagam C et al. *JAMA Network Open.*

| eTable 2. Association of risk factors with all-cause and CVD mortality (Joint effect of DR and DKD model) | | |
|---|---|---|
| **Characteristics** | **All-cause mortality** | **CVD mortality** |
| | **Multivariable*** | **Multivariable*** |
| | **HR (95% CI)** | **HR (95% CI)** |
| **Joint effect of DR and DKD** | | |
| No DR and no DKD | Reference | Reference |
| DR alone | 1.38 (1.03 – 1.86) | 1.64 (1.06 – 2.56) |
| DKD alone | 1.89 (1.40 – 2.57) | 2.26 (1.42 – 3.61) |
| Both DR and DKD | 2.76 (2.05 – 3.72) | 3.41 (2.19 – 5.32) |
| Age, per year | 1.07 (1.05 – 1.08) | 1.06 (1.03 – 1.08) |
| Gender, female | 0.59 (0.47 – 0.76) | 0.62 (0.43 – 0.89) |
| **Ethnicity** | | |
| Malay | Reference | Reference |
| Indian | 0.75 (0.57 – 0.99) | 0.81 (0.54 – 1.21) |
| Chinese | 0.80 (0.56 – 1.16) | 0.83 (0.48 – 1.43) |
| Primary/below education | 1.21 (0.91 – 1.61) | 1.05 (0.70 – 1.56) |
| History of cardiovascular disease | 1.52 (1.21 – 1.91) | 2.54 (1.85 – 3.48) |
| Current smoking | 1.11 (0.79 – 1.55) | 0.90 (0.53 – 1.53) |
| Alcohol consumption | 1.07 (0.70 – 1.66) | 0.93 (0.48 -1.78) |
| Body mass index (kg/$m^2$) | 1.00 (0.98 – 1.03) | 1.00 (0.96 – 1.03) |
| Hypertension | 0.97 (0.71 – 1.32) | 0.97 (0.62 – 1.54) |
| Serum total cholesterol (mmol/L) | 1.10 (1.00 – 1.20) | 1.01 (0.88 – 1.17) |
| Serum HDL cholesterol (mmol/L) | 0.96 (0.68 – 1.36) | 1.01 (0.60 -1.70) |
| HbA1c, % | 1.09 (1.02 – 1.16) | 1.17 (1.06 – 1.28) |

© 2019 Sabanayagam C et al. *JAMA Network Open.*

| | | |
|---|---|---|
| Duration of diabetes, year | 1.00 (0.99 – 1.02) | 1.01 (0.99 – 1.02) |

© 2019 Sabanayagam C et al. *JAMA Network Open.*
